# More Than a Small Brain: The Importance of Studying Neural Function during Development

**DOI:** 10.1523/JNEUROSCI.1367-24.2024

**Published:** 2024-11-27

**Authors:** James C. Dooley, Meike E. van der Heijden

**Affiliations:** ^1^Department of Biological Sciences, Purdue University, West Lafayette, Indiana 47907; ^2^Purdue Institute for Integrative Neuroscience, Purdue University, West Lafayette, Indiana 47907; ^3^Fralin Biomedical Research Institute at Virginia Tech Carilion, Roanoke, Virginia 24016; ^4^Center for Neurobiology Research, Roanoke, Virginia 24016; ^5^School of Neuroscience, Virginia Tech, Blacksburg, Virginia 24016

**Keywords:** cerebellum, development, motor systems, neurodevelopmental disorders, sensory systems

## Abstract

The nervous system contains complex circuits comprising thousands of cell types and trillions of connections. Here, we discuss how the field of “developmental systems neuroscience” combines the molecular and genetic perspectives of developmental neuroscience with the (typically adult-focused) functional perspective of systems neuroscience. This combination of approaches is critical to understanding how a handful of cells eventually produce the wide range of behaviors necessary for survival. Functional circuit development typically lags behind neural connectivity, leading to intermediate stages of neural activity that are either not seen in adults or, if present, are considered pathophysiological. Developmental systems neuroscience examines these intermediate stages of neural activity, mapping out the critical phases and inflection points of neural circuit function to understand how neural activity and behavior emerge across development. Beyond understanding typical development, this approach provides invaluable insight into the pathophysiology of neurodevelopmental disorders by identifying when and how functional development diverges between health and disease. We argue that developmental systems neuroscience will identify important periods of neural development, reveal novel therapeutic windows for treatment, and set the stage to answer fundamental questions about the brain in health and disease.

## Significance Statement

The brain's complexity is mirrored by the many neuroscientific disciplines working to understand its forms and functions. Systems neuroscientists aspire to understand the brain by measuring neural function and behavior, whereas developmental neuroscientists focus on molecular and genetic approaches to establish how a small population of cells develops into a mature brain. These approaches are complementary; thus, it is not surprising that these two fields have begun to intersect. This has led to the growing subfield of developmental systems neuroscience, which applies the tools of systems neuroscience to questions typically reserved for developmental neuroscience. A novel understanding of the brain's complexity is emerging from studying how the developmental trajectories of neural activity and processes produce phenotypic variability.

## Introduction

The human brain is often—and with quite a bit of hubris—referred to as the most complicated object in the universe. This complexity emerges from just a handful of cells that divide, migrate, and, ultimately, develop the connections that enable the functions and computations necessary for adaptive behaviors. Whereas this developmental process is remarkably robust, these neural functions and complex behaviors do not appear overnight. On the contrary, brain development, particularly in humans, is a protracted process (Rice and Barone, 2000; Semple et al., 2013). Yet often the steps of this developmental process are overlooked, and thus the biological underpinnings that enable the functional development of neural circuits remain elusive.

**Figure 1.**
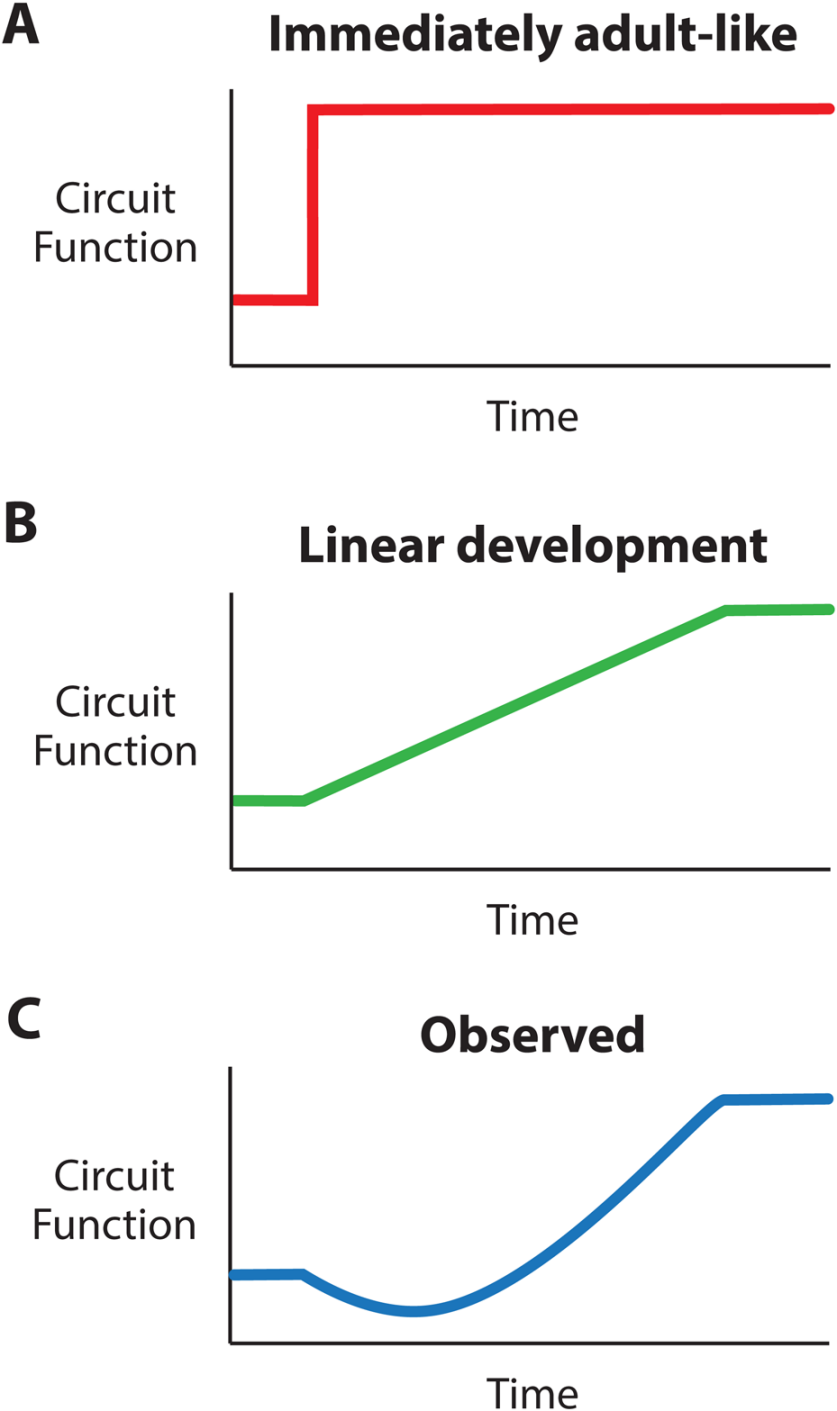
Implicit predictions of circuit function across developmental time. ***A***, Expected circuit function with no functional development. As soon as a circuit is present, it is functional. ***B***, Expected circuit function with a consistent increase across development. ***C***, Representation of observed circuit function across development, demonstrating that nonlinearities are frequently observed en route to adult-like circuit function.

Historically, the field of developmental neuroscience has used either molecular, anatomical, or genetic perspectives to understand the formation of circuits. This can be seen in developmental neuroscience textbooks: Until recently, little or no content has been devoted to how these processes translate into circuit function and behavior (Rao and Jacobson, 2005; Sanes et al., 2012). Although newer textbook editions have begun to shift this balance (Augustine et al., 2023), all too often, a limited understanding of functional development leads to implicit predictions that rarely hold up under examination. Circuit development is often taught through the lens of synaptic pruning and plasticity giving rise to the intuitive understanding that neural circuits do not show “adult-like” functionally immediately after connections develop (Fig. 1*A*). However, circuits also rarely show a consistent and stable progression toward adult-like functionality (Fig. 1*B*). Instead, on the way to an adult-like circuit, functional development follows unexpected, dynamic, and critically, nonlinear trajectories that can only be understood and appreciated when studied in detail (Fig. 1*C*). This includes periods where synaptic connectivity is established but neural activity is seemingly random (Arroyo and Feller, 2016), altogether absent (Hanse et al., 2013), or patterned in a manner that would be considered pathophysiological in adult individuals (Arancillo et al., 2015). Furthermore, many neural circuits have windows of time wherein specific events ought to happen for normal functional development, also known as critical periods. Only when the functional development of neural circuits is observed across development can we appreciate the nonlinear dynamics responsible for the complex circuits underlying our perceptions and behaviors.

In this perspective, we highlight examples of nonlinear dynamics of functional neural development based on some exemplary neural circuits in mammalian, and predominantly rodent, model organisms. We then discuss how new multidisciplinary studies combine tools from developmental neuroscience and systems neuroscience to allow discoveries about the trajectories of neural function in developmental disorders. Finally, we acknowledge some of the challenges inherent to studying developing circuits and how they can be mitigated.

## Sensory Systems

**Figure 2.**
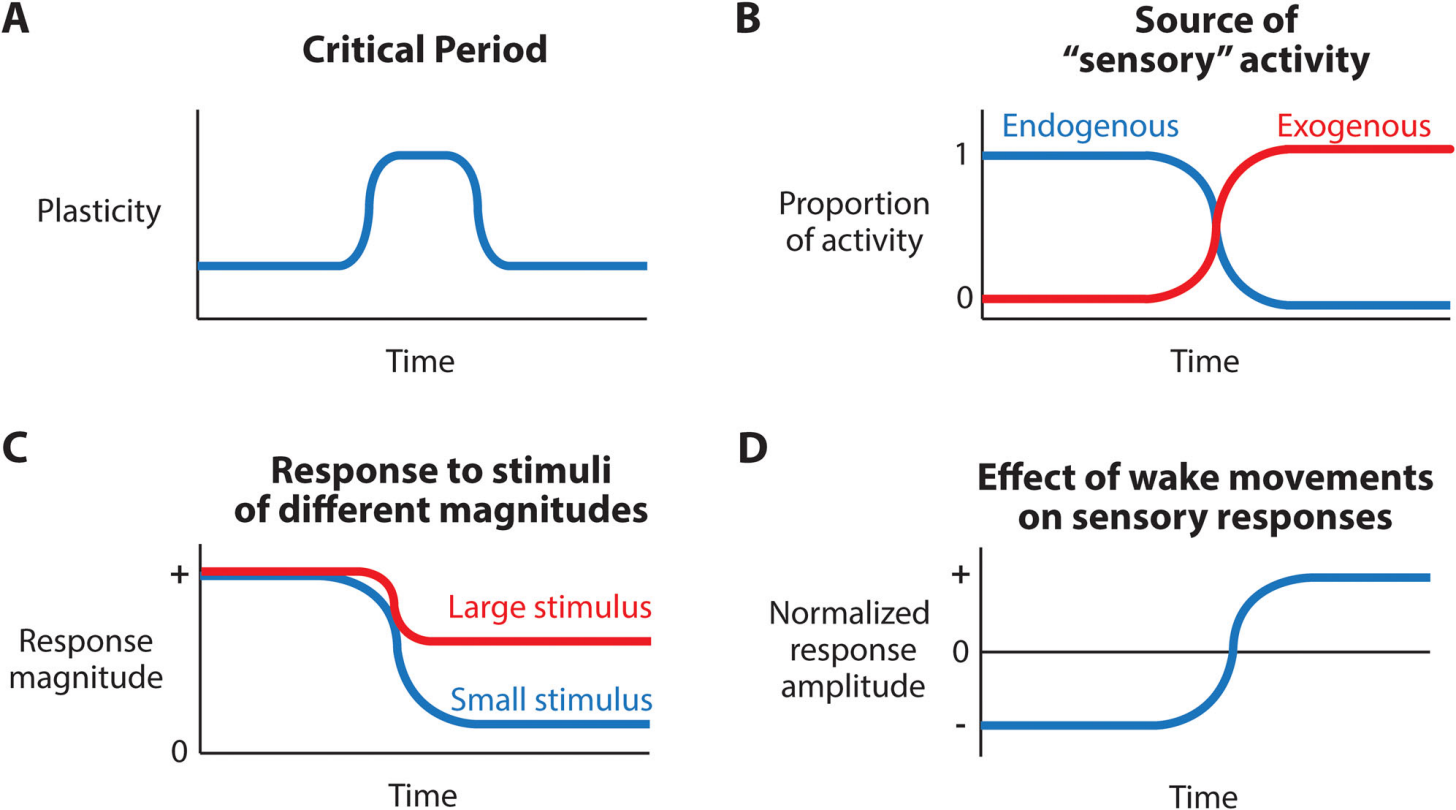
Functional changes that contribute to the nonlinear development of sensory-evoked activity. ***A***, Critical periods are a discrete window of increased circuit plasticity. ***B***, Initially, most neural activity in sensory systems is produced endogenously (i.e., retinal and cochlear waves). Eventually, neural activity in sensory systems originates from exogenous sources. ***C***, In early infancy, cortical sensory areas respond in an all-or-none fashion, with both small and large amplitude sensory stimuli giving the same response. ***D***, In early infancy, sensory responses during wake movements are inhibited. Eventually, this pattern is reversed, such that sensory responses during wake movements are enhanced.

Because the circuitry of sensory systems is often highly stereotyped and relatively well understood, neuroscientists can use sensory circuits to test whether different manipulations lead to different developmental outcomes. Consequently, understanding sensory system development is used as a model for understanding the development of the entire brain. Broadly, the development of sensory circuits follows a general pattern: Sensory systems develop dense and exuberant connections which are then pruned, resulting in the “typical” pattern of connectivity (for review, see Martini et al., 2021). While the details certainly differ depending on the circuit in question, neural activity can direct both axonal growth (Durack and Katz, 1996; White et al., 2001; Murakami et al., 2022) and the patterning of axonal pruning (Anastasiades and Butt, 2012; Antón-Bolaños et al., 2019; Guillamón-Vivancos et al., 2022). The effects of this neural activity are strongly influenced by developmentally regulated molecular mechanisms (Galuske et al., 1996). Consequently, sensory-evoked neural activity is particularly dynamic across early development, resulting in complex, nonlinear shifts in how this activity is patterned. While by no means extensive, in this section we will explore four such functional changes (Fig. 2) and discuss how these changes produce developmentally appropriate patterns of activity that help sensory systems develop but, in adults, would be pathological.

Much of the early work on the development of the visual system and visual circuits focused exclusively on molecular cues and axon guidance (e.g., the work of Robert Sperry; Sperry, 1963). Then, in the late 1960s and early 1970s, Hubel and Weisel demonstrated the existence of a critical period: Over a discrete developmental window, the left and right eyes show activity-dependent competition that establishes the size and patterning of each eye's representation in the visual thalamus and cortex (Feller, 2009; Ackman et al., 2012; Wang and Bergles, 2015). This work showed that patterned neural activity, in this case originating in the retina, can have distinct (and discrete) developmental functions (Fig. 2*A*), opening the door for future research to show how changes in the patterning of neural activity promotes different developmental outcomes. In the half-century that followed, countless researchers have identified other complex interactions between sensory activity and the development of sensory systems.

For example, consider that neurons in the developing visual and auditory systems are active before peripheral sensory receptors are responsive to light or sound. Rather than responding to external (exogenous) sensory inputs, the neurons in these sensory circuits respond to internal (endogenous) activity generated by spontaneously active neurons, which takes the form of retinal and cochlear waves (Feller, 2009; Ackman et al., 2012; Wang and Bergles, 2015). A similar phenomenon is seen in the developing sensorimotor system, via the appearance of myoclonic twitches of skeletal muscles during periods of REM sleep, only with direct activation of peripheral proprioceptors via self-generated movements (Kreider and Blumberg, 2000; Blumberg et al., 2020). We know—from work done predominantly in the visual system—that disrupting this early endogenous activity (i.e., disrupting or eliminating retinal waves) produces large changes in visual connectivity (Ackman and Crair, 2014; Arroyo and Feller, 2016; Choi et al., 2021). Of course, as development progresses, this endogenous activity decreases while exogenous sensory activity increases (Fig. 2*B*). Eventually, once exogenous activity dominates, the reappearance of endogenous activity in the retina is considered pathological, disrupting visual perception (Trenholm and Awatramani, 2015).

Focusing on the neocortex, infant cortical activity in sensory areas is strikingly discontinuous: In rats and mice, recordings in primary visual and somatosensory cortex reveal periods of silence surrounded by occasional bursts of activity (Khazipov et al., 2004; Hanganu et al., 2006; Colonnese et al., 2010). Using an electroencephalogram (EEG), this same pattern is observed in premature and perinatal humans (Whitehead et al., 2018). Despite being a typical developmental pattern, this bursty activity resembles the pathological activity seen in unconscious clinical populations, including the neocortices of deeply anesthetized and comatose individuals (Young, 2000; Amzica, 2015; Purdon et al., 2015). Of course, a key distinction is that in infants, these bursts occur alongside normal, age-appropriate behaviors, unlike the absence of behavior in unconscious clinical populations. This activity's similarity to an otherwise pathological pattern make it tempting to conclude that the burstiness seen in the infant neocortex is a byproduct of its immaturity and thus functionless. Yet this does not appear to be the case. Instead, these bursts amplify ascending sensory inputs, and this amplification is believed to strengthen thalamocortical connections (Colonnese and Phillips, 2018). The bursts result from a lack of feedback inhibition of corticothalamic circuits (Murata and Colonnese, 2016), leading to “all-or-none” cortical responses at the expense of graded responses to stimuli of different intensities (Fig. 2*C*; Colonnese et al., 2010; Dooley et al., 2020). Consequently, the resulting cortical representations of sensory inputs cannot discriminate small inputs from large inputs. Instead, these sensory-evoked bursts of activity serve a different function—enabling refinement of thalamocortical connectivity that, ultimately, enhances sensory representations. Over time, this discontinuous, bursty activity gives way to continuous neural activity and graded sensory responses, a transition that in rodents is complete by the end of the second postnatal week (Colonnese et al., 2010; Luhmann and Khazipov, 2018).

As a final example, recent studies have described a developmental change in the relationship between behavioral (sleep/wake) states and sensory activity. In adult rodents, movements during wake (e.g., locomotion) amplify sensory responses in the visual and somatosensory systems (Dadarlat and Stryker, 2017; Ayaz et al., 2019). This amplification has been shown to improve sensory discrimination (Bennett et al., 2013) and is thought to reflect attentional modulation of sensory inputs (Ferguson and Cardin, 2020). However, in early infancy, movements during wake have the exact opposite effect on sensory activity, occurring alongside a decrease in visual and somatosensory responses (Mukherjee et al., 2017; Murata and Colonnese, 2018; Dooley et al., 2020). Thus, in infant cortex, sensory inputs are dampened—or possibly altogether disregarded—during wake movements, a reversal in the function of attentional systems that, in adults, would reflect a total absence of attentional modulation. Notably, there is no such inhibition of the sensory activity occurring alongside REM sleep twitches throughout infancy. This wake-related inhibition persists until the end of the second postnatal week, when wake-related amplification begins, marking the onset of the adult-like pattern (Fig. 2*D*; Dooley and Blumberg, 2018; Murata and Colonnese, 2018). Both the mechanism and the purpose of wake-related inhibition of sensory responses in early infancy are unknown. However, as has been shown in the previous examples, it would be a mistake to reflexively assume that this phenomenon reflects circuit immaturity and thus serves no function. Rather, considering the connection between sleep and plasticity seen throughout infancy (Blumberg et al., 2022), one intriguing hypothesis is that the function of this wake-related inhibition of sensory responses is to restrict activity-dependent plasticity of sensory pathways during periods of wake.

In summary, in adults, sensory-evoked activity provides a stable representation of sensory inputs, enabling us to use our sensory systems to interact with the world. This notion is so ingrained in our conceptualization of sensory-evoked activity that it is difficult to imagine that it could serve any other function. However, during development, sensory-evoked activity often functions to promote the formation and pruning of sensory circuits and not to provide a stable representation of the world. This developmental function, although necessary for the development of sensory circuits, results in patterns of activity that fail to represent sensory inputs with “adult-like” precision. Thus, whereas in adults, these patterns would violate the basic assumption of what sensory-evoked activity “should” represent, as we have described, in infancy, they are a critical component of sensory system development.

## Motor Systems

Much like sensory systems, the developing motor system is a spontaneously active network. However, unlike sensory systems, by definition, “functional” motor structures translate neural activity into movements that can interfere with ongoing behaviors. Thus, to prevent maladaptive behaviors from nascent motor structures, different motor structures become functional—or capable of producing movement—on remarkably different timelines. Developmentally, the first movements, which occur in rodents at late embryonic ages, are directly produced in the spinal cord (Robinson et al., 2000). Work in chicks shows that these first movements are the result of spontaneous spinal activity, not sensory stimulation (Hamburger et al., 1966; Provine et al., 1970). However, because somatic and proprioceptive receptors are already functional, these movements drive activity in the developing spinal cord that creates and strengthens the first sensorimotor loops.

As development progresses, more and more motor structures become capable of movement production. In rodents, brainstem motor nuclei—including, for limb movements, the red nucleus—begin to drive ongoing behaviors around birth (Kreider and Blumberg, 2000; Del Rio-Bermudez et al., 2015). However, the developing sensorimotor network is still far from mature. In rats, the primary motor cortex (M1)—the archetype of mammalian motor structures—does not begin producing movements of its own until postnatal day 25 and does not have an “adult-like” motor map until approximately postnatal day 60 (Young et al., 2012; Singleton et al., 2021). Data from human infants who have experienced a perinatal stroke over M1 suggest a similar developmental timeline (Kirton and Deveber, 2013; also see Blumberg and Adolph, 2023a,b). In adults, damage to the motor cortex often leads to severe movement deficits or paralysis (Freund, 2007). But in infancy, brainstem motor structures can drive all the behaviors necessary for survival. Yet as infants develop, M1 eventually takes on its adult motor control functions, and as M1's motor map grows and matures, the motor maps in brainstem structures like the red nucleus appear to shrink (Williams et al., 2014; Williams and Martin, 2015).

Importantly, M1 is not silent prior to the onset of cortical motor control. Instead, its neural activity predominantly reflects sensory feedback from the movements produced in the brainstem (Chakrabarty and Martin, 2000; Tiriac and Blumberg, 2016; Dooley and Blumberg, 2018). Thus, despite its name, the activity in infant M1 closely resembles activity in the nearby primary somatosensory cortex (S1; Dooley and Blumberg, 2018; Gómez et al., 2021), a discovery that likely reflects M1 and S1's shared evolutionary origins (Kaas, 2004). Also like S1, this early sensory activity is crucial to M1's development: Chronical inhibition of M1 activity prevents the development of motor control (Chakrabarty and Martin, 2005). But chronic M1 inhibition also influences the development of other motor structures, like the red nucleus, preventing the age-appropriate decreases in motor functionality otherwise observed (Williams and Martin, 2015). This suggests that motor structures in the brainstem and cortex show activity-dependent competition, although the exact patterns of M1 and red nucleus activity that enable this competition are not yet established.

In infant rats, the sensory responses that define M1's early functional development reach M1 in parallel with S1 until the end of the second postnatal week, when the adult-like serial flow of somatosensory inputs—from S1 to M1—begins to emerge (Gómez et al., 2021). These parallel sensory inputs in S1 and M1 play a role in activating silent corticocortical synapses, a key step that determines which corticocortical connections persist, and which are eventually pruned (Anastasiades and Butt, 2012), a process that likely enables S1 and M1's somatotopic alignment. A similar pattern—including a transition from parallel to serial ascending sensory inputs—has been shown in the developing visual system (Warner et al., 2012; Murakami et al., 2022). Taken together, these findings suggest that cortical hierarchies, which are central to theories of adult neocortical function, are largely absent from infant brains, providing another example that complicates any direct comparisons of neocortical function in infants and adults.

Looking beyond M1, the prefrontal cortex is well known for its protracted development (Klune et al., 2021): In humans, the prefrontal cortex continues to develop well into young adulthood (Petanjek et al., 2011; Kolk and Rakic, 2022). Developmentally, the prefrontal cortex shares several similarities with the rest of the neocortex, showing periods of relative silence, punctuated by bursts of activity (Chini et al., 2022; Kalemaki et al., 2022) that are patterned by sleep and wake (Gómez et al., 2023). But unlike other cortical areas that largely have a single, dominating input (Colonnese et al., 2010; Dooley and Blumberg, 2018; Glanz et al., 2021), the prefrontal cortex's early activity is patterned by inputs that are heterogeneous and elusive (Gómez et al., 2023). The prefrontal cortex's protracted development and relative independence from sensory inputs enable different environments to shape its higher-order, decision-making functions (Johnson et al., 2015; Werchan and Amso, 2017). Whereas this makes the prefrontal cortex quite adaptable, in some environments, it can lead to adult dysfunction (Bitzenhofer et al., 2020; Kolk and Rakic, 2022). Thus, as with the sensory and motor systems, identifying the inputs that drive early the prefrontal cortex activity and understanding how different environments shape the prefrontal cortex's functional development are critical to understanding its function and dysfunction in adults.

## Cerebellum

Thus, neural circuit function does not develop on a linear trajectory in the neocortex, where information is predominantly encoded through rate codes and communicated via long-range connections that are established during early development. Is this also true for the cerebellum, a subcortical brain region that encodes information through a combination of rate and synchrony, where the major cell types fire continuously, and where dynamic circuit rewiring occurs postnatally?

The cerebellum's development is different from other brain regions in multiple aspects. Its predominant cell type, the granule cell, continues to proliferate for several weeks after birth in rodents, and for several years after birth in human (Wallace, 1999; Limperopoulos et al., 2005; Volpe, 2009). With the integration of granule cells, the glutamatergic afferent connections into the cerebellum get rearranged and pruned (van der Heijden and Sillitoe, 2021). Unsurprisingly, neural function and cerebellum-dependent behaviors are still developing during this period—but similar to cortical regions, this development does not occur linearly.

The cerebellar circuitry does not develop equally fast across all regions and the acquisition of specific behaviors partially follows the temporal dynamics of the anatomical development of the cerebellar lobule's mediating these behaviors (Beekhof et al., 2021). Nevertheless, the cerebellum is important for the normal acquisition of neonatal motor reflexes (negative geotaxis and righting reflexes) even in the second postnatal week (van der Heijden, Gill, et al., 2022; van der Heijden et al., 2023), well before the cellular or synaptic circuitry is fully established anywhere across the cerebellar cortex (van der Heijden and Sillitoe, 2021). This suggests that even an immature cerebellum, whose circuitry differs greatly from the adult cerebellum, still contributes to early postnatal behaviors.

This early contribution of the cerebellum to behaviors is especially puzzling given the pattern of neural activity in the cerebellum. In the adult cerebellum, Purkinje cells, the cerebellum's primary projection neurons, fire at a high and continuous rate (50–100 Hz; Heck et al., 2013; van der Heijden, Brown, et al., 2022). In younger rodents, not only is the Purkinje cell neural firing rate slower, but it is also hallmarked by intermittent pauses, resulting in a burst-like firing pattern (Arancillo et al., 2015; Beekhof et al., 2021; van der Heijden et al., 2021). In adult animals, however, burst-like patterns are predominantly observed in animal models for cerebellar motor disorders: Purkinje cell bursts are often considered a hallmark for pathophysiological cerebellar function (Snell et al., 2022; van der Heijden et al., 2024). Thus, “normal” function should be considered within a specific developmental context, as the same neural signals may be differentially encoded at distinct time points.

This developmental context for neural coding is indeed confirmed by a recent paper that shows that neural signals about self-movement are differentially encoded in the cerebellum during development compared with adulthood (Richardson et al., 2024). The transient states of neural activity in the cerebellum across development further raise the question: Does this early neural activity serve to establish behavior or circuit function during specific critical periods, much as is observed in neocortical brain regions? While the existence of critical periods in the cerebellum is still an ongoing area for exploration, several studies suggest that neural activity (Badura et al., 2018) and plasticity (Wu et al., 2024) are differentially important for the establishment of cerebellar behaviors and are different developmental time points. This suggests that activity-dependent changes in neural circuit development also occur in the cerebellum. Further research is necessary to elucidate the precise developmental mechanisms establishing adult function and cellular underpinnings that differentiate between cerebellar circuit function between neonatal and adult animals.

## Neurodevelopmental Disorders

**Figure 3.**
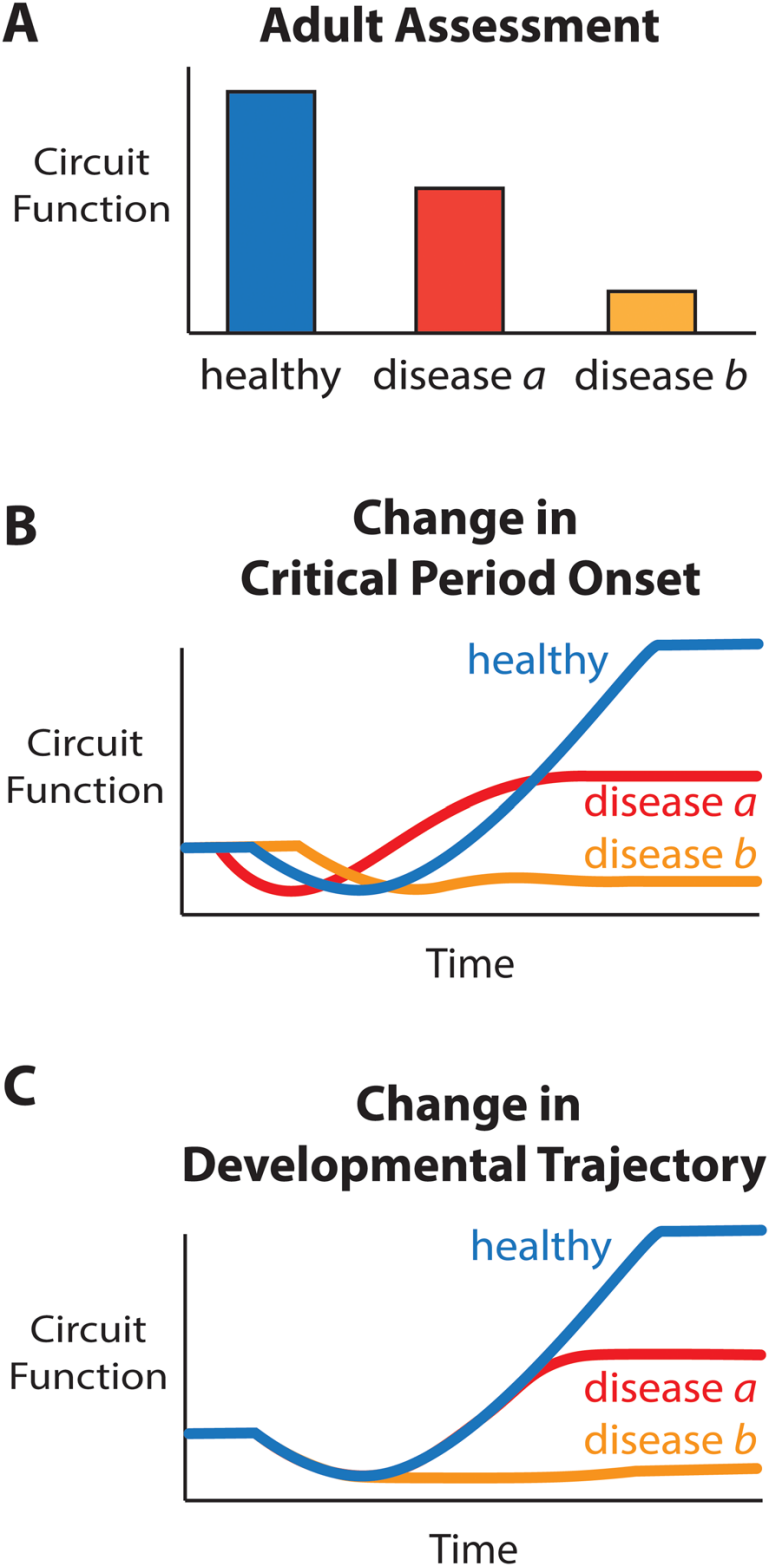
Advantages of a developmental perspective on circuit function. ***A***, Circuit function (and dysfunction) is apparent in adults, but assessing circuit function at a single time point fails to provide perspective on the trajectory to dysfunction. ***B***, ***C***, Examples of possible routes to the decrease in circuit function observed in “disease a” and “disease b” shown in ***A***.

In medical practices, pediatric specialists take care of patients during infancy, childhood, and adolescence. This specialization reflects the fact that differences in diseases, physiology, and anatomy between children and adults that evolve during development warrant specialized care (National Academies of Sciences, Engineering, and Medicine, 2023). In contrast, at the bench, the investigation of neurodevelopmental disorders is not always approached from a developmental angle. Consequently, in animal models for neurological disease, investigations of neural function are often only performed in adult animals. These studies provide insight into the final, static difference in neural function between animals that underwent healthy development and those that did not (Fig. 3*A*). Nevertheless, such studies do not provide insight into when the developmental divergence occurred or why this divergence results in abnormal neural function. For example, a change in onset of developmental critical period could miss the critical window for future events and thereby stall out at a certain developmental stage (Fig. 3*B*). Alternatively, the developmental trajectory may track that of healthy circuit developmental until a certain point at which the circuit no further develops in the disease state (Fig. 3*C*). These changes in developmental dynamics in disease context would require different therapeutic interventions, and therefore careful investigation of developmental dynamics is essential when studying neurodevelopmental disorders.

Understanding the timing of the divergence between healthy and disordered development is essential for therapeutic interventions. Oftentimes, the question of timing is addressed by studying the period when neural activity or gene function is necessary for functional development (Krishnan et al., 2015; Li et al., 2021; Gibson et al., 2022; Hunter et al., 2024). Identification of these critical periods has strong implications for when therapeutic interventions, such as gene-replacement therapies, would be most effective. However, the studies into critical periods do not fully address why neural activity or gene function is required during a specific period of neural development. A better understanding of the dynamic processes that produce critical periods will provide a deeper mechanistic insight into the steps that occur toward building the complex adult brain.

Indeed, these benefits are not merely theoretical. In various experimental paradigms, combining genetic perturbations with electrical recording techniques has uncovered changes in neural activity that would not have been identified—or even predicted—from genetic analysis alone (Nomura et al., 2017; Camp et al., 2023; Hussein et al., 2023; Ciceri et al., 2024). Such mechanistic studies may uncover developmental dynamics that are essential for neural function in adulthood (Tasnim et al., 2024). Common changes in these functional dynamics may explain why a wide array of genetic perturbations can result in similar neurodevelopmental phenotypes in infants. Combining both developmental and systems neuroscience approaches allows the creation of therapeutic interventions reliant on precisely timed neuromodulation. Such therapies—treating the underlying abnormal neural activity rather than genetic perturbation—could be applied to a broader range of patients than highly personalized genetic therapies.

Finally, studying genetic models of neurodevelopmental disorders in the context of development will provide invaluable opportunities to start linking information about gene function to neural function. Many different genetic mutations are linked to neurodevelopmental disorders with similar features (Zech et al., 2020; Gidziela et al., 2023). Conversely, different mutations in the same gene or incomplete penetrance can cause a range of neurodevelopmental phenotypes associated with the same gene (Dzinovic et al., 2022; de Masfrand et al., 2024). Studying how genetic mutations influence neural function may bridge the gap in the understanding of how genotype influences phenotype and opens up novel avenues to treat a growing population of individuals with neurodevelopmental disorders.

## Challenges

Studying the functional development of neural circuits presents several challenges. To start, many behavioral assays used to study neural function are specified for adult animals, and these assays often rely on movements and behaviors that are limited in younger rodents. These limitations can be partially overcome by studying age-specific or enriched behaviors, like twitching during sleep, developmental motor reflexes, or early postnatal vocalizations. However, compared with studying behavior in adult animals, there is a dearth of assays suitable to test behavior in developing rodents. Expanding and validating behavioral assays to assess neural function during development would be highly valuable to the field of developmental systems neuroscience.

There are significant limitations concerning the technical approaches used to study neural function in developing animals. Stable neural recordings require recording devices that are securely attached to both the brain and the skull. In adult animals, this stability is typically achieved by cementing the recording device to the skull or securing the animal's skull in a rig, as the skull and brain maintain a static relationship. However, in developing rodents, the ongoing growth of the skull and brain poses challenges for securing recording devices over extended periods (Gottlieb et al., 1977; Khazipov et al., 2015). Furthermore, the soft composition of the growing skull complicates efforts to stabilize the brain in a recording or surgical rig. Finally, rodents are often single-housed after the attachment of recording devices or headplates, which is not feasible for early neonatal pups that are still nursing. These technical limitations can be addressed through customized surgical techniques, specialized recording platforms (Blumberg et al., 2015), and optimized anesthetic regimens, although these solutions require continued refinement and validation.

In addition, the changing dynamics during development inherently require the testing of neural function at multiple time points. There are two ways to achieve this: (1) longitudinal studies, which necessitate overcoming the above challenges, or (2) acute experiments performed in different cohorts of animals at numerous time points. These acute experiments require more resources than a single time point study, and relatively larger sample sizes are needed to have sufficient statistical power to compare developmental dynamics across multiple time points. Intentional experimental designs are necessary to optimize the number of measurements necessary to capture developmental dynamics in neural function. While resource intensive, studying multiple time points is necessary to fully understand the temporal dynamics of neural development and the underlying mechanisms for the establishment of circuit function.

Despite technical and experimental hurdles in developmental systems neuroscience, the field also benefits from recent technical innovations in both developmental neuroscience and systems neuroscience. Organoid studies now allow the investigation of functional neural properties in the context of cocultured neurons in a human genetic background. Although organoids present their limitations, particularly concerning behavior, these studies have already found fundamental differences in the developmental dynamics between healthy developing and neurodevelopmentally impaired organoid cultures (Hussein et al., 2023; Li et al., 2023). Additionally, the ever-expanding list of experimental approaches of systems neuroscience, including novel viral constructs, optogenetics, and calcium imaging, can be readily applied in developing systems, opening up a plethora of novel approaches to address existing questions. Thus, more than ever before, the scientific toolbox is well suited to study developmental systems neuroscience.

## Conclusion

Behavioral development is sometimes characterized as a continuous increase in behavioral complexity. The earliest movements are simple twitches, which are used as building blocks for multijoint limb coordination, locomotion, and eventually, complex, adaptive behaviors (Altman and Sudarshan, 1975; Richmond and Sachs, 1980; Hall and Oppenheim, 1987; Robinson et al., 2000). This pattern, from simple to complex, can also be observed in the development of receptive fields. In the cortex, visual receptive fields are initially simple and homogeneous, but as development progresses, they become complex and more heterogeneous (Rochefort et al., 2009; Colonnese et al., 2010). A similar trajectory is also observed in developing auditory and somatosensory cortices (Kral and Pallas, 2011; Seelke et al., 2012; Glanz et al., 2023). Perhaps because of these developmental progressions, implicitly, we imagine that functional development is a journey from simple to complex patterns of activity and that this process is straightforward and predictable (Fig. 1*A*,*B*). But, as discussed throughout this perspective, functional development is rarely straightforward. And even with perfect molecular and genetic knowledge, functional development is still not predictable.

Understanding how nervous systems develop necessitates understanding how anatomy, molecular factors, genetics, and neuronal activity all interact to promote neural functions and complex behaviors. Each of these elements is a dimension in and of itself that should not be ignored. However, the functional development of circuits does more than expand our knowledge of typical development: It is also fundamental for understanding the mechanisms that give rise to neurodevelopmental disorders. Studying differences in adult circuit function provides insight into the downstream consequences of these disorders. However, overreliance on adults makes it difficult—or even impossible—to establish when functional development diverges from normal dynamics and whether the adult dysfunction is reflective of abnormal developmental processes, compensatory mechanisms, or a combination thereof (Fig. 3). Knowing when in development healthy and pathological states diverge in terms of neural function, perception, and behavior is crucial for both the development of new therapies and, in some instances, the timing at which these therapies are applied. The timing of therapeutic interventions is especially important when functional circuit development is reliant on specific events occurring during critical windows.

Until recently, our understanding of nervous system development was predominantly defined by molecular, anatomical, and genetic methods, as recording neuronal activity in unanesthetized infant animals was not possible. But this is no longer the case—dozens of labs around the world routinely record neural activity from behaving infant animals (Pocratsky et al., 2023; Fechner et al., 2024; Reid et al., 2024; Vita et al., 2024). Although it remains challenging, developmental systems neuroscience is now a viable and fruitful approach. And unlike many subspecialties, in developmental systems neuroscience, some of the most foundational data has not yet been collected. Observation is the first step of the scientific method; a phenomenon needs to be documented before it can be manipulated. Yet even to date, fully descriptive studies of functional development continue to yield surprising and impactful results (Cai et al., 2024), illuminating wholly unknown—and heretofore unpredicted—developmental trajectories.
